# Flavonoids Reduce Lipid Peroxides and Increase Glutathione Levels in Pooled Human Liver Microsomes (HLMs)

**DOI:** 10.4236/abc.2021.116019

**Published:** 2021-12-15

**Authors:** William Yaw Boadi, Camille Stevenson, Dontrez Johnson, Mohamed Adel Mohamed

**Affiliations:** 1 Departments of Biological Sciences, Tennessee State University, Nashville, USA; 2 Departments of Chemistry, Tennessee State University, Nashville, USA

**Keywords:** Flavonoids, Glutathione (GSH), Human Liver Microsomes (HLMs), Lipid Peroxidation, Oxidative Stress

## Abstract

The effects of each of the flavonoids; genistein (G), quercetin (Q) and kaempferol (K) at several doses on lipid peroxides (LP) and reduced glutathione (GSH) in pooled human liver microsomes (HLMs) were investigated following the oxidative damage for 4, 6, 18 and 24 hr. HLMs (1 mg/ml) were exposed to each of the above flavonoids at 0, 5, 10, 15, 20 or 25 μM and incubated for the respective times as previously stated. Our hypothesis was that HLMs exposed to the flavonoids for the respective exposure times can decrease LP and increase GSH in HLMs to better cope with the oxidative stress. The results of our studies indicate that each of the flavonoids significantly (p < 0.01) decreased LP compared to their respective controls. The highest decrease in LP was observed for K followed by Q and G. Significant increases (p < 0.01) in GSH were observed for the flavonoid doses tested with the highest levels observed for Q for the 24-hr. incubation. The findings suggest that the flavonoids modulate oxidative stress in HLMs by decreasing LP and such decreases in LPs may be due to the increasing and or the replenished levels of GSH in the said cells to better cope with the oxidative stress.

## 1. Introduction

Lipid peroxidation is a complex process occurring in aerobic cells and reflects the interaction between molecular oxygen and polyunsaturated fatty acids. Radicals are known to take part in lipid peroxidation, which causes food deterioration, aging organisms and cancer promotion [1] [2]. These reactive oxygen species (ROS) are reported to be involved in asthma, inflammation, arthritis, neurodegeneration, Parkinson’s disease, mongolism and perhaps dementia [3] [4]. Antioxidants act as radical scavengers, inhibit lipid peroxidation and other free radical-mediated processes, thereby protecting the human body from several diseases attributed to reactions involving ROS [5] [6]. It has been reported that various phenolic antioxidants, such as flavonoids, tannins, coumarins, xanthones and more recently procyanidins scavenge ROS dose dependently, and thus are viewed as promising therapeutic drugs for ROS pathologies [7] [8] [9] [10].

Glutathione (GSH) has been reported as a potent endogenous antioxidant that helps to protect cells from several noxious stimuli including ROS [11]. Furthermore, several investigators have suggested that stress stimulates lipid peroxidation in several tissues which may also cause GSH reduction [12] [13] [14] [15] [16].

In our previous and recent studies, the effects of the flavonoids, genistein (G), kaempferol (K), and quercetin (Q) on phospho tensin homolog (PTEN) levels in cancer cells (i.e., breast (BT549) and lung (A549)), human embroyonic kidney cells (HEK293), and levels of TBARS in peripheral blood mononuclear cells (PBMCs) were respectively investigated [17]. The results indicate that the single treatments of the cells with either G, K or Q increased total PTEN levels in a dose-dependent manner as well as in TBARS in PBMCs. Thus, consumption of foods containing polyphenols may help reduce the causes of factors related to the metabolic syndrome [18] [19] [20] and those associated with the anti-inflammatory mechanisms and improved antioxidant capacity [21] [22].

It has been reported that human liver microsomes (HLMs) provide the most convenient way to study cytochrome polymorphic (CYP)-mediated metabolism [23]. Microsomes are a subcellular fraction of tissue obtained by differential high-speed centrifugation [24]. As to how the flavonoids modulate the oxidative damage and GSH levels in HLMs are limited or non-existent. Thus, the purpose of the present studies was to investigate the single treatments of genistein (G), kaempferol (K) and quercetin (Q) at 0, 5 10, 15, 20, and 25 μM doses on TBARS and GSH in HLMs before and after the oxidative damage [17] [25] [26] [27]. Our studies sought to test the following hypotheses: 1) that, exposure of HLMs to either G, K or Q can decrease TBARS in those cells and better cope with oxidative stress. 2) That decreases in TBARS in HLMs following the exposure may be due to the concomitant increases in the intracellular levels of GSH. The proposed studies represent an effort to define how G, K and Q modulate TBARS, a tumor promoter, [25] and GSH, a naturally occurring antioxidant, in HLMs cells following exposure to those insults over time. In terms of future directions, we will follow the lead set by the experimental results.

## 2. Materials and Methods

### 2.1. Chemicals

Isoflavone kaempferol (3,5,7-trihydroxy-2-(4-hydroxyphenyl)-4H-1-benzopyran-4-one, 98% purity), and genistein (4’,5,7-trihydroxy isoflavone, 98%), and quercetin dihydrate (3,3’,4’,5,7-Pentahydroxyflavone dihydrate, 98%) were from Sigma-Aldrich (St. Louis, MO). Human liver microsomes (HLMs) from pooled individual human donors’ liver, ferrous chloride tetrahydrate (FeCl_2_∙4H_2_O, purity, 99%), phosphate-buffered saline (PBS, 0.1 M), dimethyl sulfoxide (DMSO, 99% purity) and H_2_O_2_ were purchased from Fisher Scientific Suwanee, GA. Double deionized water was purified using a Milli-Q system (Millipore Corporation, MA).

### 2.2. Preparation of Standard and Stock Solutions

The flavonoids were dissolved in DMSO. The respective solutions were further diluted (between 50 – 100 times, depending on the dose needed) with DMSO before adding to the incubation mixture. The final DMSO concentration in the incubation mixture was 0.05% v/v [17].

### 2.3. Treatment of HLMs with the Flavonoids

A typical incubation mixture was prepared in a total volume of 200 μl with HLMs (1 mg/ml), with the respective single treatments, G, K or Q at 0, 5, 10, 15, 20, and 25 μM respectively. All the controls and samples following the treatments were cultured at 37°C for 4, 6, 18 and 24 hr. respectively.

### 2.4. Antioxidant Activity Testing of Flavonoids in HLMs

Antioxidative activity as described by [28] was used with some modifications. HLMs (1 mg/ml) in a total reaction volume of 200 μl of PBS were incubated with Fe^2+^ (50 μM) ions [29] and H_2_O_2_ (0.01 mM) with and without flavonoid sample at 0, 5, 10, 15, 20 and 25 μM for each of the three flavonoids and were tested in this study for 4, 6, 18 and 24 hr. respectively at 5% CO_2_.

### 2.5. Reagents Effect on HLMs Cell Growth and Viability

HLMs were treated in separate experiments to determine whether DMSO (0.05%), H_2_O_2_ (0.01 mM) and Fe^2+^ (50 μM) ions were affecting cell growth and viability that might compromise the levels of TBARS and GSH. HLMs (1 mg/ml) per tube were incubated respectively with DMSO, H_2_O_2_, and Fe^2+^ ions, as stated above for 24 hr. Cell viability and number were assessed as we have previously reported [26] [27].

### 2.6. Preparation of Samples after Treatments with the Flavonoids

Following the incubations, samples were centrifuged in a refrigerated Eppendorf table-top centrifuge (Model # 5804 R, Suwanee, GA) at 4°C for 10 min at 3000 RPM. HLMs following the incubations were rinsed with sterile phosphate buffered saline (PBS) to remove any reagents and prepared as previously reported [17].

### 2.7. Analysis of Lipid Peroxides in HLMs

The method as previously reported [17] was used with some slight modifications. TBARS standard curves were used to determine the levels of lipid peroxides in the samples.

### 2.8. Analysis of Reduced Glutathione (GSH) in HLMs

Reduced GSH was analyzed as described [30] with some modifications as prescribed by the Calbiochem GSH Assay Kit (No. 354102). Following the incubation, cells centrifuged at 3000 ×g for 10 min at 4°C in a refrigerated table top Eppendorf centrifuge (Fisher Scientific, Suwanee GA) and resuspended in 300 μl of 5% metaphosphoric acid (MPA). Cells were sonicated under ice for 30 seconds using a cell dismembrator (Fisher Scientific, Suwanee GA) at a setting of 3. The homogenate was then centrifuged at 3000 ×g for 10 minutes at 4°C. The resulting supernatant was removed and stored at −70°C until used for the analysis of GSH. GSH levels in cells were calculated from the standard curve using GSH prepared in 5% MPA (freshly prepared) as standard and expressed in μM.

### 2.9. Statistical Analysis

Results are expressed as means of ±SD. Statistical significance was determined as previously described (Boadi *et al*. 2014). Each value in all figures represents the mean for each dose of the single flavonoid and TBARS and GSH tested, which was assayed in triplicates.

## 3. Results

### 3.1. Effects of Exposure of Flavonoids on Lipid Peroxides in HLMs for 4, 6, 18 and 24 Hr

**Figure 1.**
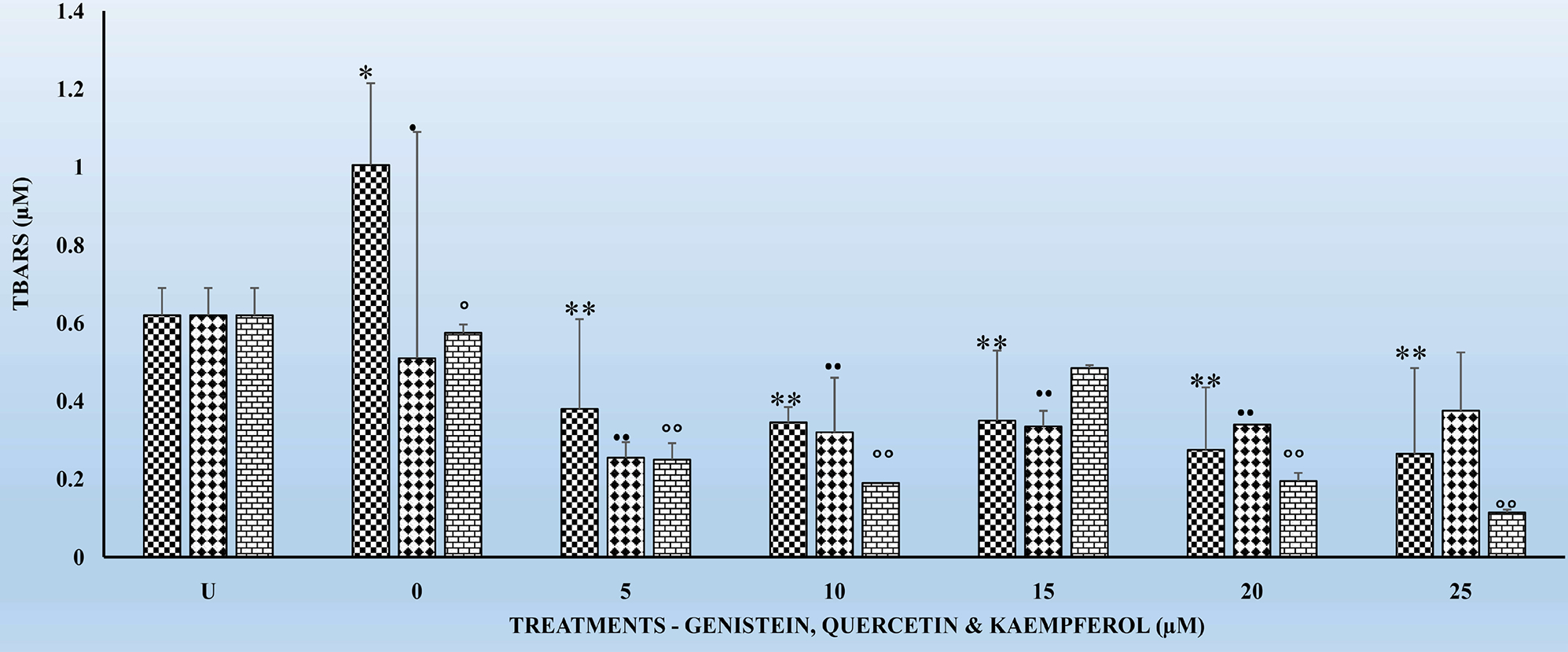
The effects several doses (0, 5, 10, 15, 20, and 25 μM) each of genistein (G), quercetin (Q) and kaempferol (K) on thiobarbituric reactive substances (TBARS) in pooled human liver microsomes (HLMs), following incubation at 37°C and 5% CO_2_ for 4 h. Each bar chart for each flavonoid ± standard deviation in this and other figures (*i.e*., Figures 2–4) in this article represent mean the for 3 different experiments for each dose level of G, Q and K tested and, which was assayed in triplicates. Statistical significances denoted by asterisk in Figures 2–4 are shown as comparison between the respective control (i.e., without G, Q and K) and G, Q and K HLMs treated subgroups. Vertical bars in this and other figures denote standard deviation. The X-axis labels for Figures 2–4 are defined as follows: U means untreated cells samples; 0 means control cell samples not treated with G, Q and K; 5, 10, 15, 20, and 25 μM means cell samples were each treated with the respective dose of G, Q and K for 4, 6, 18 and 24 h.

**Figure 2.**
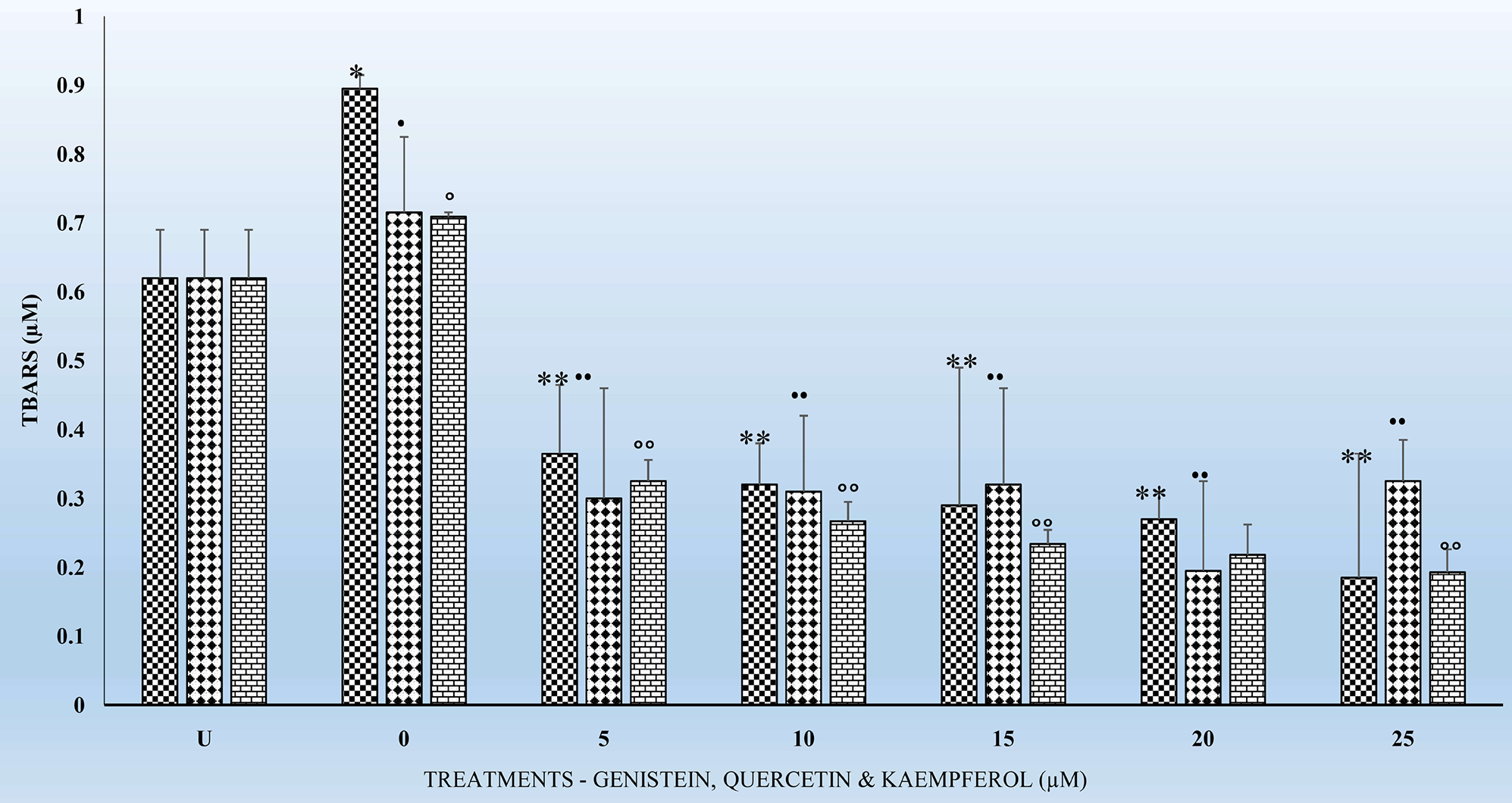
The effects several doses (0, 5, 10, 15, 20, and 25 μM) each of G, Q and K on TBARS in HLMs, following incubation at 37°C and 5% CO_2_ for 6 h. For comparison and statistical differences see Figure 1.

**Figure 3.**
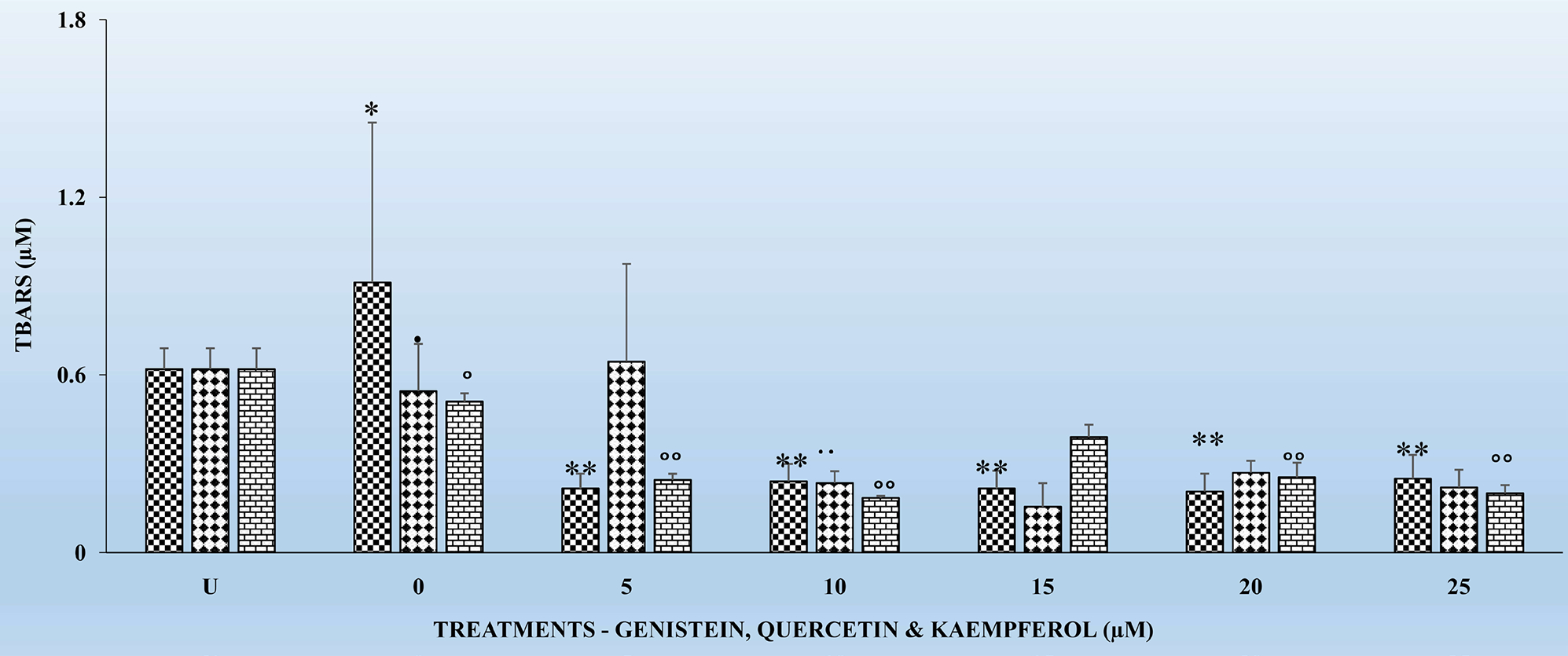
The effects several doses (0, 5, 10, 15, 20, and 25 μM) each of G, Q and K on TBARS in HLMs, following incubation at 37°C and 5% CO_2_ for 18 h. For comparison and statistical differences see Figure 1.

**Figure 4.**
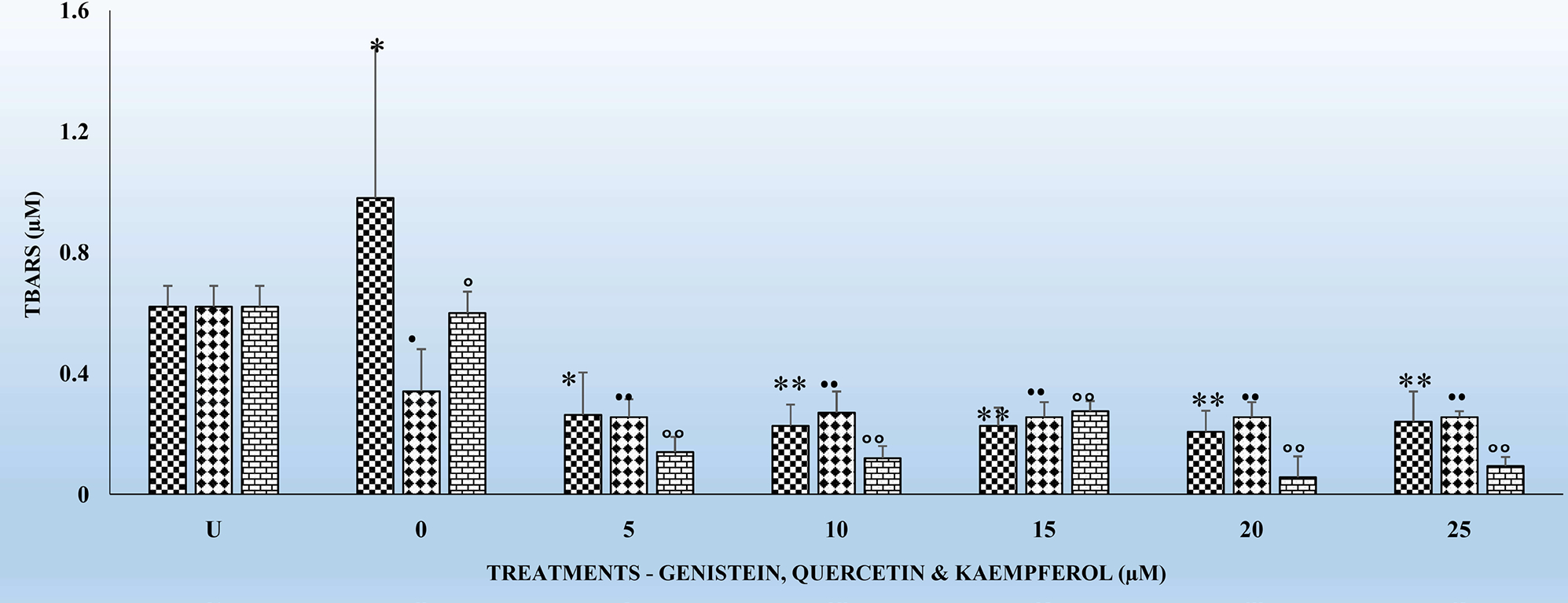
The effects of several doses (0, 5, 10, 15, 20, and 25 μM) each of G, Q and K on TBARS in HLMs, following incubation at 37°C and 5% CO_2_ for 24 h. For comparison and statistical differences see Figure 1.

Figures 1–4 show the effects of the respective exposure of K, G and Q on TBARS in HLMs following the respective incubation times.

### 3.2. Effects of the Acute Exposure of Flavonoids on TBARS in HLMs for 4 and 6 Hrs

Figure 1 and Figure 2 show the respective TBARS in HLMs for G, Q and K treatments for the 4 and 6 hr incubation periods. TBARS remained the same for the untreated (U) HLMs. However, TBARS decreased significantly (p < 0.01) for all the 3 flavonoids tested in comparison to their respective controls. The decreases in TBARS were higher for K treated samples followed by G and Q. A similar significant decrease (p < 0.01) in TBARS was observed for the 6 hr. treatments (Figure 2). However, the decreases in TBARS were not dose-dependent (Figure 1 and Figure 2). Significant decreases in TBARS were observed at the 5 μM for all the flavonoids irrespective of the incubation period. In comparing the decreases in TBARS for the 4 hr. (Figure 1) to that of the 6 hr. (Figure 2), K decreased TBARS more compared to either G or Q for the respective time periods.

### 3.3. Effects of the Chronic Exposure of Flavonoids on TBARS in HLMs for 18 and 24 Hr

Figure 3 and Figure 4 show the effects of the respective exposure of the flavonoids on TBARS in HLMs following incubation for 18 and 24 hr. As previously observed for the 4 and 6 hr. incubation periods, TBARS, remained the same for the untreated (U) HLMs samples. However, TBARS decreased significantly (p < 0.01) for each of the flavonoids tested in comparison to their respective controls. The significant decreases (p < 0.01) in TBARS for the 24 hr. treatments (Figure 4) were higher compared to the 18 hr. Significant (p < 0.05) decreases in TBARS were observed as early at the 5 μM for all the respective flavonoids. K again as previously observed for the 4 and 6 hr. (Figure 1 and Figure 2) decreased TBARS more compared to G and Q for the above time periods.

### 3.4. Effects of Flavonoids on GSH Levels in HLMs Following Incubations for 4, 6, 12 and 18 Hr

Tables 1–3 show the effects of the single treatments of G, Q and K 0, 5, 10, 15, 20 and 25 μM on GSH levels in HLMs following oxidation by the Fenton’s pathway and incubation for 4, 6 18 and 24 h respectively. GSH levels increased (p < 0.05) for G (Table 1) at the 10 – 25 μM; Q and K (Table 2 and Table 3) at the 5 – 25 μM doses compared to their respective controls and were time dependent. Levels of GSH were sustained and replenished during the oxidative damage for the Q, G and K treated samples in comparison to their respective controls. Despite the significant increases (p < 0.05) in GSH for Q at the said doses, each of the flavonoids were capable of replenishing GSH levels in cells to combat the stress. On comparative basis Q appears to have the greatest effect in increasing GSH levels, followed by G and K.

### 3.5. Relationship between Lipid Peroxides and GSH Levels in HLMs Following the Exposure to the Flavonoids

One of the hypotheses in this study was to test whether there is any correlation between exposure of HLMs to the flavonoids for 6, 12, 18 and 24 hr. can offer better protection to HLMs through decreased TBARS and increased and replenished GSH over time. It is interesting to report that treatments of HLMs to flavonoids and after the oxidative damage were as effective in significantly (p < 0.05) reducing TBARS (Figures 1–3) as well as significantly increasing (p < 01) GSH levels (Tables 1–3) over time. The decreases in TBARS as well as the increases in GSH were highest for Q followed by K and G.

## 4. Discussion

### 4.1. Lipid Peroxides and Flavonoids as Antioxidants

ROS formed in cells, including singlet oxygen, can oxidize several cellular constituents like lipids, proteins and DNA. Lipid oxidation occurs in membranes, especially in the intracellular ones. During lipid peroxidation many different products, e.g. aldehydes, peroxides and ROS are formed. The direct effect of lipid peroxidation is the membrane destruction. Secondary effects include the interaction of lipid peroxidation reaction products with other cellular components, e.g. the reaction of aldehydes with DNA, which can result in mutagenicity or carcinogenicity [31] [32]. The protective effects of flavonoids have been attributed to a wide variety of mechanisms, including modulating enzyme activities resulting in the decreased carcinogenicity of xenobiotics [27] [32] [33].

### 4.2. Studies Purpose and Hypothesis

As to how the flavonoids modulate the mechanisms of the levels of TBARS following the oxidative damage in HLMs have not been well studied or very non-existent in the literature. Thus, the purpose of the present study was two-fold. First, to investigate the effects of exposure of HLMs to each of G, K and Q at 0, 5 10, 15, 20, and 25 μM on lipid peroxides measured as, TBARS. And second, to study if there is any correlation between the levels of LPs and GSH following the exposure to these flavonoids and the oxidative damage. Our studies sought to test the following two hypotheses: 1) that, exposure of HLMs to either G, K or Q can decrease TBARS in those cells and to better cope with oxidative stress. 2) That, exposure of HLMs to either G, K or Q can help replenish GSH in those cells and better cope with oxidative stress. The HLMs model was selected because it serves as a good system to measure antioxidant activity and are close to the *in vivo* situation where both aqueous and lipid phases are present [34].

### 4.3. Effects of Genistein, Kaempferol and Quercetin on Lipid Peroxides

The findings in this study indicate that, TBARS, decreased significantly (p < 0.01) for each of the flavonoids in comparison to their respective controls (Figures 1–4). The decreased TBARS as observed in the current studies is similar, to that reported by Menéndez *et al*. [35]. In that studies, the authors observed inhibition of rat microsomal lipid peroxides by the oral administration of D002, a compound, made up of a mixture of higher primary alcohols purified from bee wax. The authors attributed the inhibition in the lipid peroxides to the actions of superoxide dismutase (SOD), catalase and glutathione peroxidase (GSH-Px) which represent an efficient defense system against hazards of lipid peroxidation [35].

### 4.4. Relationship between Lipid Peroxides and Glutathione

We have observed increased levels of GSH for all the tested flavonoids (Tables 1–3) following the oxidative stress. The above seem to suggest that these compounds must have helped the HLMs to regenerate enough GSH to offset the oxidative stress and probably through the GSH-Redox system. Increases in GSH in the present studies lend support to our previous studies where we demonstrated that the flavonoids can upregulate the GSH-Redox system thereby reducing the damaging effects of ROS [30]. Such observations are like those by Menéndez *et al*. [35] who attributed that to the actions of SOD, catalase and GSH-Px against hazards of lipid peroxidation. On the contrary, studies by Wattenberg *et al*. [36] on the CYP system have shown different modulatory effects of flavonoids activity both *in vitro* and *in vivo*. The authors suggested that exposure of cells to flavonoids might have caused the induction of either phase I or phase II enzymes that can result in increased detoxification of carcinogens [36] [37]. We are currently conducting similar experiments to substantiate if there is any correlation between the increased GSH and that of some phase I or phase II enzymes following exposure of the HLMs to the flavonoids that might partly have contributed to the decreased TBARS.

### 4.5. Effects of the Incubation Periods on Lipid Peroxides

It is also very interesting to know that significant (p < 0.01) decreases in TBARS were observed at the 5 μM for the tested flavonoids irrespective of the incubation period (Figures 1–4). The above observation is very interesting and at variance with what we have seen in our previous studies in other cell types [17] [27] [32] where decreases in TBARS were time- and dose-dependent. The only plausible explanation for such observations in HLMs may be three-fold. First, it is possible that the effects of flavonoids on enzymes are generally dependent on the concentrations of the flavonoids present. Second, the bioavailability of the different flavonoids in the cells may be low which not reflect the concentrations tested under *in vitro* conditions [33]. Furthermore, the HLMs that we have used in the current studies were from pooled human donors. Thus, the marked interindividual variability in drug-metabolizing enzymes as result of genetic and other environmental factors [33] might have attributed to our current observations. Nevertheless, the antioxidant activities of flavonoids against metal-induced lipid peroxidation are probably a consequence of their metal-chelating and free radical scavenging activities [38].

## 5. Conclusion

We have employed single flavonoid treatments G, Q and K at different concentrations to reduce the levels of TBARS and to increase GSH levels in HLMs. By using the single treatments of these flavonoids over time the cellular and biological activities based on the decreased TBARS and increased intracellular levels of GSH would be useful for developing health supplements and/or therapeutic drugs against diseases associated with the oxidative damage.

## Figures and Tables

**Table 1.** GSH Levels (μmoles/mg protein) in HLMs following exposure to genistein for 4, 6, 18 and 24 hr. respectively. Each cell for each flavonoid ± standard deviation in this and other tables (*i.e.*, Table 2 and Table 3) in this article represent mean the for 3 different experiments for each dose level of G tested and, which was assayed in triplicates. Statistical significances denoted by letters, in Table 1 are shown as comparison between the respective control (*i.e.*, without G) and GHLMs treated subgroups for the respective incubation periods.

Incubation	0	5	10	15	20	25 (μM)

4	1.15 ± 0.16	3.41 ± 0.41	3.11 ± 0.24^a^	3.69 ± 0.38^a^	2.19 ± 0.24^a^	4.07 ± 0.24^a^
6	1.29 ± 0.03	4.74 ± 0.99	3.98 ± 0.50^b^	5.78 ± 0.46^b^	4.79 ± 0.87^b^	5.34 ± 1.32^a^
18	1.10 ± 0.12	3.40 ± 0.11	3.24 ± 0.20^cd^	3.19 ± 0.60^bc^	3.18 ± 1.15	6.80 ± 0.75^b^
24	1.07 ± 0.05	4.57 ± 0.21	5.74 ± 0.19^d^	3.92 ± 0.34^bd^	4.14 ± 0.62^c^	7.14 ± 0.54^b^

**Table 2.** GSH Levels (μmoles/mg protein) in HLMs following exposure to quercetin for 4, 6, 18 and 24 hr. respectively. For comparison and statistical differences see Table 1.

Incubation	0	5	10	15	20	25 (μM)

4	1.11 ± 0.16	3.41 ± 0.41^a^	3.51 ± 0.24^a^	3.69 ± 0.38^a^	4.99 ± 0.24^a^	4.07 ± 0.24^a^
6	1.09 ± 0.03	4.74 ± 0.99	4.98 ± 0.50^b^	6.78 ± 0.46^b^	9.79 ± 0.87^b^	11.34 ± 1.32^b^
18	1.11 ± 0.12	5.40 ± 0.11^b^	6.24 ± 0.23^c^	6.19 ± 0.63^b^	10.48 ± 1.15^c^	12.80 ± 0.75^c^
24	1.17 ± 0.05	6.57 ± 0.21^c^	7.74 ± 0.19^d^	7.92 ± 0.34^c^	11.14 ± 0.62^d^	14.14 ± 0.54^d^

**Table 3.** GSH Levels (μmoles/mg protein) in HLMs following exposure to kaempferol for 4, 6, 18 and 24 hr. respectively. For comparison and statistical differences see Table 1.

Incubation	0	5	10	15	20	25 (μM)

4	1.15 ± 0.16	3.41 ± 0.41^a^	3.51 ± 0.24^a^	3.69 ± 0.38^a^	2.99 ± 0.24^a^	4.77 ± 0.24^a^
6	1.29 ± 0.03	4.74 ± 0.99	5.98 ± 0.50^b^	5.78 ± 0.46^b^	6.79 ± 0.87^b^	8.34 ± 1.32^b^
18	1.11 ± 0.12	1.40 ± 0.11^c^	1.24 ± 0.23^c^	2.19 ± 0.63^c^	3.48 ± 1.15^bc^	2.80 ± 0.75^bc^
24	1.27 ± 0.05	1.57 ± 0.21^d^	1.74 ± 0.19^d^	1.92 ± 0.34^d^	3.14 ± 0.62^c^	14.14 ± 0.54^d^
